# Supplementary material for: A novel method for generating public keys involving matrix operations
Source: PLoS One. 2024 Oct 30;19(10):e0312690. doi: 10.1371/journal.pone.0312690 (PMC11524494; doi:10.1371/journal.pone.0312690)
Supplement: Attachment — Submitted filename: Response to Reviewers.pdf [file pone.0312690.s001.pdf]

# Reply to reviewers

Thank you for your valuable suggestions and comments. We hope our changes can meet the reviewer's requirements and lead to the publication of the manuscript.

**Response to Reviewer #1**

**[Comment 5]** Check the reference are cited correctly.

**[Answer]** Thank you for pointing out such kinds of problems. We checked carefully to avoid such mistakes, please see the highlighted portion in Page 8. We also checked for errors in the Reference.

**Response to Reviewer #3**
**[Comment 1]** The abstract of the paper is too brief, and it is suggested to add some content.
**[Answer]** We expanded the Abstract to make it more detailed.
**[Comment 2]** Table headings are recommended for the top of the table, not the bottom.
**[Answer]** We have revised. Please see three tables in the manuscript.
**[Comment 3]** The discussion about recent related schemes...
**[Answer]** We added some related schemes and cited the relevant paper. Please see the highlighted portion in Page 2.
**[Comment 4]** In the conclusion part, it is suggested to add the expression of future work.
**[Answer]** We outlined our future plans. Please see the last paragraph in Section 5.
**[Comment 5]** It is suggested that the author increase the function and performance comparison with other schemes.
**[Answer]** We compared our scheme with IBC, TF-CPK and SM2 implicit certificates. Our scheme has the lowest computation cost. Please see Section 4 in the manuscript.
**[Comment 5]** References should not appear in the abstract.
**[Answer]** We have deleted these two citations.

We have found and corrected some clerical errors throughout the manuscript.
Once again, thank you for your kindly help!
